# Identifying and recommending validated measures to assess depression and anxiety outcomes in the field of arts and health

**DOI:** 10.3389/fpsyg.2025.1628292

**Published:** 2025-09-03

**Authors:** Nik Ilya, Jean C. J. Liu, Kat R. Agres

**Affiliations:** ^1^Centre for Music and Health, Yong Siew Toh Conservatory of Music, National University of Singapore, Singapore, Singapore; ^2^Centre for Evidence and Implementation, Singapore, Singapore; ^3^Yong Loo Lin School of Medicine, National University of Singapore, Singapore, Singapore; ^4^Yale-NUS College, National University of Singapore, Singapore, Singapore

**Keywords:** arts and health, evaluation, mental health, validated scales, recommendations

## Abstract

**Introduction:**

Interest in the use of arts-based interventions to improve mental health outcomes has been emerging in healthcare and community settings. However, rigorous evaluation and research on the effectiveness of these interventions are still limited. Various resources have been published to encourage an expansion and improvement in the quality of the evidence base in this intersectional field. Yet, many of these resources either stop short of recommending validated outcome measures or provide justifications when they do so, nor do they identify the prevalence of use of these outcome measures. We provide practitioners in the field with recommended measures, identified through a prevalence analysis in the literature, and the associated considerations that practitioners should consider when choosing these scales.

**Materials and methods:**

We conducted a two-part literature review to identify depression and anxiety scales suitable for arts and health interventions. The review begins by identifying canonically validated tools used in medical and health research to document depression and anxiety symptoms. The second part of the review documents existing usage rates of each scale in studies involving arts and health interventions across 18 arts domains to provide a state of the field regarding the use of validated tools to assess mental health outcomes.

**Results:**

A total of 44 depression and 81 anxiety scales were identified from 31 review papers. The Hospital Anxiety and Depression Scale (HADS) and the Beck Depression Inventory (BDI) emerged as the most widely used scales for measuring depression symptoms. The State–Trait Anxiety Inventory (STAI) was identified as the most frequently used scale for measuring anxiety symptoms.

**Discussion:**

We found that the BDI was primarily used to evaluate performing arts interventions, the STAI was used for performing and visual art interventions, and the HADS was widely used across all arts domains. Our findings highlight key measures for the field, and we provide recommendations for their use, supporting arts and health practitioners in moving toward more rigorous evaluation methods.

## 1 Introduction

Mental health is a significant and growing public health issue. Globally, mental health conditions account for an estimated 16% of disability-adjusted life years, resulting in a considerable economic impact that costs approximately 4–8% of gross domestic product at the regional level (Arias et al., 2022). In response, the World Health Organization (WHO) has called for a concerted effort to improve the mental well-being of individuals and communities and to enhance the capacity to help those in need of support (Singh, 2021).

Arts-based initiatives have increasingly been implemented as a way to improve mental health outcomes [e.g., as seen in efforts such as social prescribing (Morse et al., 2022) and arts-on-prescription (Bungay et al., 2023) initiatives]. In addressing the holistic needs of patients, medical practitioners have been shifting away from a biological view of health toward a biopsychosocial model (Borrell-Carrió et al., 2004), placing greater emphasis on the social determinants of health (Andermann and CLEAR Collaboration, 2016). Additionally, arts and cultural activities are increasingly being recognized as a low-cost, non-pharmaceutical, and scalable means of supporting the health and well-being of individuals and communities (Fancourt and Finn, 2019; Jensen and Bonde, 2018), and arts and health initiatives, such as arts-on-prescription, can alleviate the burden on healthcare systems (Stuckey and Nobel, 2010).

Studies on arts and mental health have reported positive outcomes from arts activities, which suggest that the arts do indeed have the potential to make an impact on the growing burden that healthcare systems face, but most of these results stem from individual studies (Stuckey and Nobel, 2010). Larger reviews of the literature have underlined that the field lacks methodological rigor and an analytical framework (Napierala et al., 2022), while systematic reviews and meta-analyses have also highlighted a need to develop a core set of measures to build a stronger evidence base (Smith et al., 2021) and allow for comparative research (Bickerdike et al., 2017; Cooper et al., 2022). These issues have resulted in difficulties for arts activities being integrated into mainstream evidence-based public health campaigns and impede artists’ efforts in receiving funding. Although there have been concerted efforts from funding bodies and arts organizations in various countries such as the US (Exec Order No. 14084, 2022; Blair, 2024) and the UK (Fancourt et al., 2020) to address these issues, a need to increase the scale and quality of research and evaluation is still vital to close these gaps.

However, arts practitioners also face several challenges in improving the quality of their evaluations, as a wide range of contexts and environments exist in the field. Venues for arts programs encompass auditoriums for theater shows and concerts, community settings where dance or choir groups meet, and beyond. The timeframes of these interventions are also generally shorter than many clinical interventions, which may span months, as many art programs are much shorter. These contexts may not always be amenable to traditional clinical scales, which are usually administered in more controlled settings or by trained surveyors. While several toolkits have recently emerged (e.g., Creative Scotland, 2025; Daykin and Joss, 2016; Warran et al., 2025, and so on) that target these gaps and aim to support expanding the evidence base for arts and health initiatives, most do not offer specific recommendations for outcome measures or scales. In the few that do, the rationale and justification for these choices are often missing, potentially leading users to adopt measures that may not be ideal for evaluating their specific activities and intended effects and limiting comparability and replicability across studies. Furthermore, the Core Outcome Measures in Effectiveness Trials (COMET) initiative database for health research still lacks an entry for the arts and health field. As a result, the field of arts and health lacks specific guidance not only on *which* outcomes should be measured but also, importantly, on *how* practitioners who may not have received training in conducting evaluation might choose the tools to do so. This would help them better understand the effects of their initiatives in a manner that more closely resembles traditional health research and allow for the formal dissemination of results to acquire funding for their initiatives.

We aim to support practitioners in addressing these challenges and bridging the gap in the arts and health field. Here, we conduct a review of the prevalence of scales by first identifying the most widely used scales to assess depression and anxiety symptoms. We then examine their properties and assess their suitability for implementation in the context of arts for health activities to provide recommendations for arts and health practitioners. Finally, we review the usage of validated measures across prominent arts domains to gain a snapshot of the research maturity of these domains within the Arts and Mental Health research.

Based on this review, we provide recommendations for validated scales to aid practitioners in the arts and health field who may not be well-versed in research or outcome evaluation, enabling them to conduct these evidence-gathering activities independently. Additionally, this review feeds into a larger Arts and Health evaluation toolkit being developed to support the field through recommendations of scales for a variety of health outcomes, the associated concerns when making these decisions, as well as best practices when conducting outcome evaluations (Agres et al., 2025).

This review distinguishes itself from existing toolkits and frameworks by offering specific recommendations for validated scales to assess depression and anxiety symptoms in the context of arts and health, along with practical guidance based on common contexts and program designs. Another distinguishing feature of this work is that it reports an explicit methodology for identifying and recommending appropriate scales, which is rarely found in existing resources (which are few and seldom make specific recommendations or provide a rationale for prioritizing certain measures over others).

### 1.1 Overview of paper

In the first part of this study, we examine evaluation measures of mental health, with a focus on depression and anxiety as the outcome measures of interest due to their prominence and importance to mental health. Additionally, to cater to arts and health practitioners, we will focus on tools used in public health settings that are appropriate for use with general populations and groupings of data, rather than clinical conditions, which are usually targeted for use with individuals in specific clinical groups (e.g., individuals on the autism spectrum). To achieve this, we examined review papers from the top 10 psychiatry and mental health journals over a 10-year period (2013–2023) on PubMed to gather all the measurement tools included, then excluded tools not appropriate for use in general populations and non-clinical settings. We then identified the most commonly used measures by the frequency of mentions of each measurement tool within the review papers that surfaced in our literature review.

Subsequently, in the second section of our study, we examine how frequently the top measures identified in our literature review are used across various domains of arts and culture, such as music, theater, visual arts, and so on, to better understand the state of these fields in the literature and to identify which of these domains have more published research and have been using validated scales to measure these mental health outcomes. We achieved this by examining the popularity of each scale across 18 arts and cultural domains to determine which scales have been most frequently used in published works over a 10-year period (2013–2023). We conclude this paper with our recommendations based on the popularity and characteristics of these top measures, along with suggestions for the use of these evaluation tools by arts practitioners and the different settings they operate under, compared to the relatively more controlled settings of most medical evaluations. The factors we considered when making recommendations include:

1. Training is required to administer the scale—scales that require less training are preferred, as many arts practitioners may not have expertise or experience with conducting evaluations.
2. Target demographic of the scale—scales that can be used in the general population or community samples and are validated for these uses, i.e., scales that are not tailored for use with specific clinical populations.
3. Length (number of items, duration) of the tool.
4. Cost of licensing and usage—as arts practitioners may not receive funding for their activities, we highlight cost-effective scales that allow for rigorous evaluation without sacrificing accuracy and validity.
5. Whether it is possible to administer the scale multiple times in a particular time window (e.g., for analysis of impact using a pre- and post-test approach).

As many arts and health evaluations and research utilize a pre- and post- or repeated measures design, we also wish to highlight that, in addition to the factors listed above, the recall period of a scale—which refers to the timeframe the scale is concerned with evaluating, such as 1 week—is also the minimum duration between administrations of the scale in any data collection schedule (i.e., a 1-week recall period would mean that required administrations should be at least 1 week apart). Study design is highly relevant, as many arts and health evaluations imply a pre- and post- or repeated measures design. Additionally, the validity and reliability of scales are also psychometrically tested according to their recall periods (Stull et al., 2009). Researchers/practitioners should always take the recall period into account when designing and evaluating their interventions.

We focus on depression and anxiety, as they are two of the more commonly diagnosed mental health conditions (National Collaborating Centre for Mental Health, 2011; World Health Organization, 2022) and are becoming common targets for community-based arts interventions. As many validated and established instruments are available, practitioners wanting to evaluate the outcomes of their activities must make consequential decisions at an early stage of their evaluation process regarding which outcome measures to use. Since there is no singular “best” tool for all applications, we make recommendations based on the considerations listed above to guide practitioners and better inform their decision-making. The structure of the rest of the paper is as follows: First, we provide the methods used for our two-part literature search on the prevalence of scales and lay out our criteria for determining the most popular scales. Next, we discuss the results of our literature search and provide a brief background on these scales, along with a handful of important characteristics to consider when selecting a scale for use. We then present the results of the second portion of our literature search, which illustrates the popularity of the identified scales across 18 arts domains, and highlight some patterns of usage within the literature. Finally, we discuss our findings and make recommendations for practitioners.

## 2 Materials and methods

### 2.1 Measures search

For the first part of this paper, PubMed was selected as the main database due to its focus on biomedical literature and open access format. Initial searches were performed on PubMed to retrieve review papers published within the past 10 years (from 2013 to 2023), as we wanted to focus on tools that were more likely to be currently in use in the published literature. Thereafter, we sifted through all papers retrieved from the search and extracted all tools relevant to our outcome measures, omitting tools that were primarily focused on other outcomes, including transdiagnostic constructs or other indicators of the outcome measures (i.e., the Automatic Thoughts Questionnaire for depression). To keep the number of records retrieved in our initial search at a manageable level, the search was further limited to the top 10 journals of 2022 in the Psychiatry and Mental Health subject category, as ranked by SCImago. This list included World Psychiatry, Lancet Psychiatry, Annual Review of Clinical Psychology, JAMA Psychiatry, Evidence-Based Mental Health, Psychotherapy and Psychosomatics, Clinical Psychology Review, American Journal of Psychiatry, Journal of the American Academy of Child and Adolescent Psychiatry, and Lancet Healthy Longevity. SCImago was used as the journal ranking source, as it is one of the leading journal indexing platforms in the world. The 10 journals were gathered from SCImago’s Psychiatry and Mental Health category because they represent the top journals in the field and constituted the most recent list available at the time the search was performed.

The search strategy used in the first part of this paper included ((((“Outcome Measure”[Title/Abstract]) AND ((“2013/01/01”[Date - Publication]: “2023/12/31”[Date - Publication]))) AND (“review”[Title/Abstract])) AND ((Questionnaire[Title/Abstract] OR Measure[Title/Abstract] OR Scale[Title/Abstract] OR Tool[Title/Abstract] OR Instrument[Title/Abstract]))) AND (“World Psychiatry” OR “Lancet” OR “Annual Review of Clinical Psychology” OR JAMA Psychiatry” OR “Evidence-based mental health” OR “Psychotherapy and Psychosomatics” OR “Clinical psychology review” OR “American Journal of Psychiatry” OR “Journal of the American Academy of Child and Adolescent Psychiatry” OR “Lancet Healthy Longevity”[Journal]).

All review articles from the search were considered, and we extracted all tools and measures used, assessing them based on five criteria:

1. More than one mention of the tool within the list of review papers extracted
2. The tool is available in the English language
3. The tool does not require a clinician or medically trained personnel to administer
4. The tool is applicable to the general population/not a specific clinical population
5. The tool is not created or tailored to pediatric, adolescent, or older adult populations

**Figure 1:**
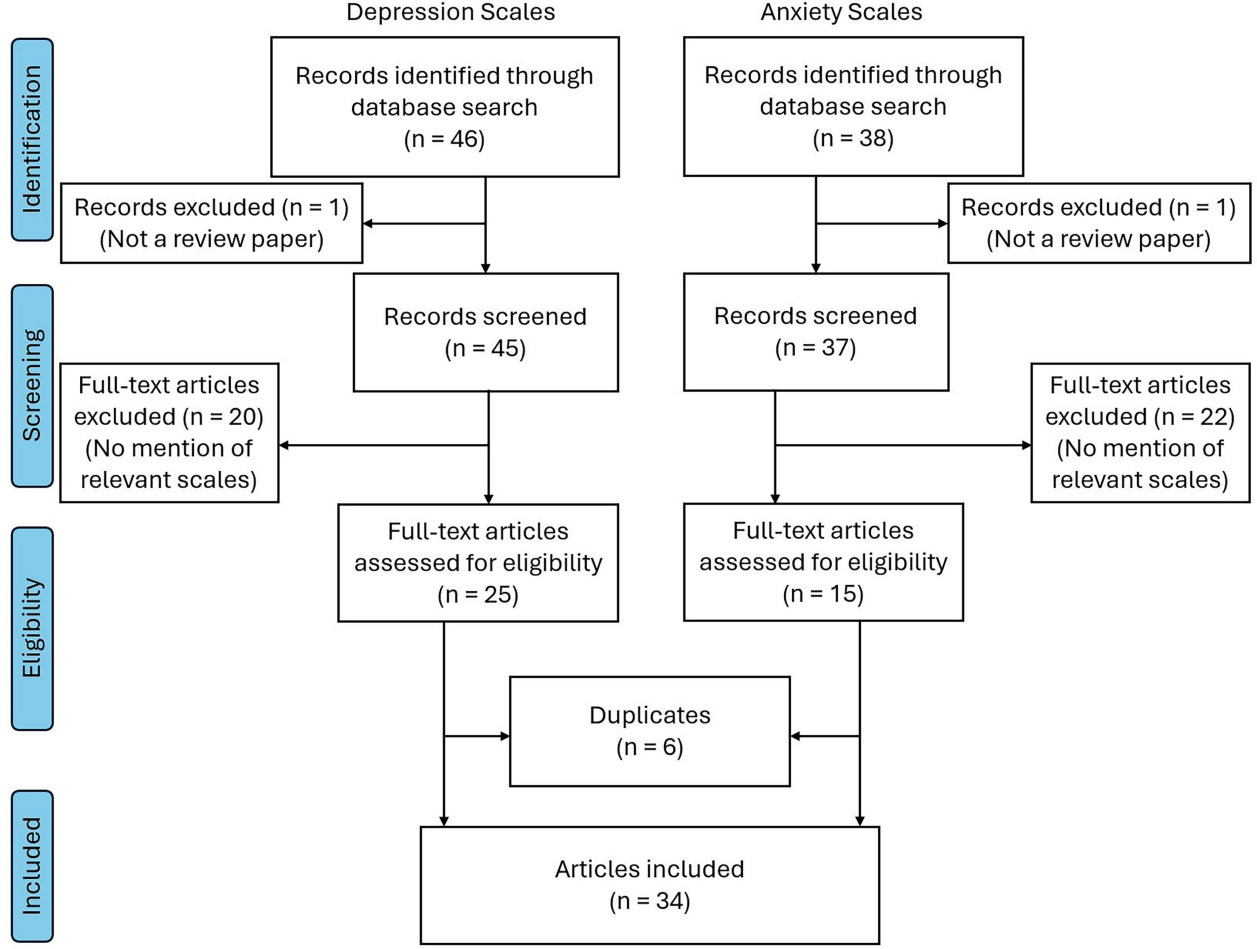
PRISMA flow diagram.

Since this effort is focused on identifying measures to help arts and health practitioners build their capacity for evaluating their activities and programs, our selected inclusion criteria focus on making the administration of such scales accessible to artist practitioners. Additionally, we exclude tools that are created or tailored for pediatric, adolescent, or older adult population samples. As we aim to recommend a small handful of validated scales for use by arts and health practitioners and to narrow the scope of our review in the second part of this paper, we selected the top five scales for each outcome to further evaluate their suitability for use by arts and health practitioners or researchers (Figure 1).

#### 2.1.1 Depression review: identification of depression scales

Our depression review was conducted in accordance with the method described above. We searched the PubMed database for review papers focusing on depression using a specified set of terms to extract measurement instruments.

From this search, we gathered a total of 46 records, and among these, 25 articles were relevant to our review. Within these 25 articles, a total of 44 questionnaires measuring depression were extracted, with 12 questionnaires meeting our four inclusion criteria. We then selected the top 5 questionnaires to further evaluate and focus on for the second part of this paper.

To confirm the validity of the depression scales we extracted from our literature search, we referred to the “*Handbook of Clinical Rating Scales and Assessment in Psychiatry and Mental Health*.” Three depression scales highlighted in this textbook were present in our literature review.

#### 2.1.2 General popularity of each depression tool

We investigated the popularity of each of the 12 questionnaires in the three databases outlined above (i.e., PubMed, Google Scholar, and HaPI), focusing on any published work and not limiting the searches to review papers. In addition, we noted the frequency with which each scale was mentioned in the 25 articles included in our review.

We began our general searches of each tool in the PubMed database, using the name of the questionnaire as the search term in quotes, i.e., “Hospital Anxiety and Depression Scale.” We followed up on this by using the same search term in the HaPI database as another indicator of popularity within a broader range of disciplines. Using PubMed and Google Scholar, we assessed the popularity of each of the 12 scales across the 18 arts activities of interest (Table 1) by searching for the name of each scale in combination with each arts activity. For example, one such search was “Hospital Anxiety and Depression Scale” AND “Music.”

**Table 1:** Characteristics of shortlisted depression and anxiety scales.

Scale	Time to administer	No. of items	Levels of measurement	Recall period	Cost
Beck Depression Inventory (BDI)	10 min	21	4-point ordinal scale (range 0–3)	The past 2 weeks	Copyrighted
Center for Epidemiologic Studies Depression Scale (CES-D)	20 min	20	4-point ordinal scale (range 0–3)	The past week	Free
Hospital Anxiety and Depression Scale (HADS)	2–5 min	14	4-point ordinal scale (range 0–3)	The past week	Permission required, English version is usually free for research and clinical use
Patient Health Questionnaire-9 (PHQ-9)	2–5 min	9	4-point ordinal scale (range 0–3)	The past 2 weeks	Free
Depression, Anxiety, Stress Scale-21 (DASS-21)	5–10 min	21	4-point ordinal scale (range 0–3)	The past week	Free for research and clinical use
Beck Anxiety Inventory (BAI)	5–10 min	21	4-point ordinal scale	Past month	Copyrighted
Generalized Anxiety Disorder Scale (GAD-7)	10–15 min	7	4-point ordinal scale (range 0–3)	Past 2 weeks	Free
State–Trait Anxiety Inventory (STAI)	10–20 min	40	4-point ordinal scale (range 1–4)	SA: current TA: 2 months	Copyrighted

These searches allowed us to create a map illustrating the popularity of each scale in each database and to make comparisons between scales within the scientific domains covered by each database.

#### 2.1.3 Anxiety review: identification of depression scales

Our anxiety review was also conducted using the same method described previously, using the PubMed database to search for review papers related to anxiety measures. The search term for this was: ((“anxiety”[Title/Abstract] AND (“questionnaire”[Title/Abstract] OR “scale”[Title/Abstract] OR “measure”[Title/Abstract] OR “tool”[Title/Abstract] OR “instrument”[Title/Abstract]) AND “review”[Publication Type] AND 2013/01/01:3000/12/12[Date - Publication] AND (“review”[Publication Type] AND 2013/01/01:3000/12/12[Date - Publication])) AND ((review[Filter]) AND (2013/1/1:3000/12/12[pdat]))) AND (“World Psychiatry” OR “Lancet Psychiatry” OR “Annual Review of Clinical Psychology” OR JAMA Psychiatry” OR “Evidence-based mental health” OR “Psychotherapy and Psychosomatics” OR “Clinical psychology review” OR “American Journal of Psychiatry” OR “Journal of the American Academy of Child and Adolescent Psychiatry” OR “Lancet Healthy Longevity”[Journal]).

From this search, we gathered a total of 38 articles. Of these, 18 were relevant and yielded a total of 30 questionnaires measuring anxiety, with four questionnaires meeting our inclusion criteria. Since an inadequate number of questionnaires met our inclusion criteria, all four questionnaires were selected for further evaluation in the second part of our paper.

To confirm the validity of the most commonly used anxiety scales, we referred to the “Handbook of Clinical Rating Scales and Assessment in Psychiatry and Mental Health.” Four anxiety scales highlighted in this textbook appeared in our literature review.

#### 2.1.4 General popularity of each anxiety tool

We investigated the popularity of the four anxiety questionnaires in the same three databases outlined above and used in the depression review. We also focused on searching for published articles, not limited to review papers, and noted the frequency with which each scale was mentioned in the 18 papers retrieved from our earlier search of review papers.

As done previously, we also began our general searches of each anxiety tool in the PubMed database, following the same method for search terms and then proceeding similarly with the HaPI database. We then followed the same procedure for investigating each scale’s popularity in arts studies on PubMed and Google Scholar, creating a heat map for each database.

### 2.2 Frequency of use of scales in arts domains

For the second part of this paper, we chose to perform three further searches with the qualifying tools to investigate whether these scales have been previously used in published arts-related interventions in the last 10 years. We used the name of the tool as one of the search terms within each of the arts domains contained in the scope of this paper. Search terms focused on arts activities were gathered from sources such as Fancourt and Finn (2019) scoping review published by the WHO’s regional office for Europe and align with art forms identified in Sonke et al. (2023) paper defining arts participation. These terms include performing arts, visual arts, music, dance, crafts, choir, theater, painting, choral, film, electronic arts, concerts, poetry, gallery, singing, literary festivals, museums, and arts.

The first search to identify previously used tools in arts-based studies was conducted on PubMed. Next, Google Scholar was chosen due to its extensive coverage across many disciplines to identify whether other arts-related studies using these tools had been published and to ascertain how popular these tools were in the overall scientific literature, specifically in arts-related activities. Lastly, we conducted searches in the Health and Psychosocial Instruments (HaPI) database, which covers disciplines related to health and social sciences, such as medicine, public health, and psychology, among others. This database serves as a repository for researchers to locate measurement tools that may be relevant for their research. For us, HaPI was used to confirm the results of our literature review.

Additionally, we performed two general searches on PubMed and Google Scholar to understand the popularity of these tools in the medical domain and the wider scientific literature, respectively. For this, we used the name of each qualifying tool as the search term, e.g., “Beck Anxiety Inventory.”

Recording the number of results from each of our searches, we uncovered how popular each measurement tool was in different scientific domains and arts activities. We examined any discrepancies in the usage of specific tools between these areas of interest.

We also highlight one limitation that may be inherently present in this methodology—scales that measure multiple outcome measures may yield higher results compared to a single outcome measure scale. However, this may not inaccurately represent a scale’s popularity.

#### 2.2.1 Cross-referencing

To ensure the comprehensiveness and validity of the scales identified in our review, we cross-referenced our findings with a well-established medical textbook in the field. This additional step was taken to confirm that the scales we identified are recognized and highlighted in authoritative academic sources. This reference focused on measurement and diagnostic tools in psychiatry and mental health that are currently being taught to medical students. The textbook used for this was the “*Handbook of Clinical Rating Scales and Assessment in Psychiatry and Mental Health,”* edited by Baer and Blais (2010).

#### 2.2.2 Popularity of depression and anxiety scales in the art/heritage domain

The literature search on PubMed and Google Scholar for the popularity of the shortlisted depression and anxiety scales was conducted across various art activities listed above. The arts activities included “Performing Arts,” “Arts,” “Music,” “Dance,” “Theater,” “Singing,” “Visual Arts,” “Crafts,” “Painting,” “Literary Festivals,” “Poetry,” “Electronic Arts,” “Gallery,” “Museum,” “Choir,” “Choral,” “Film,” and “Concert.”

**Figure 2:**
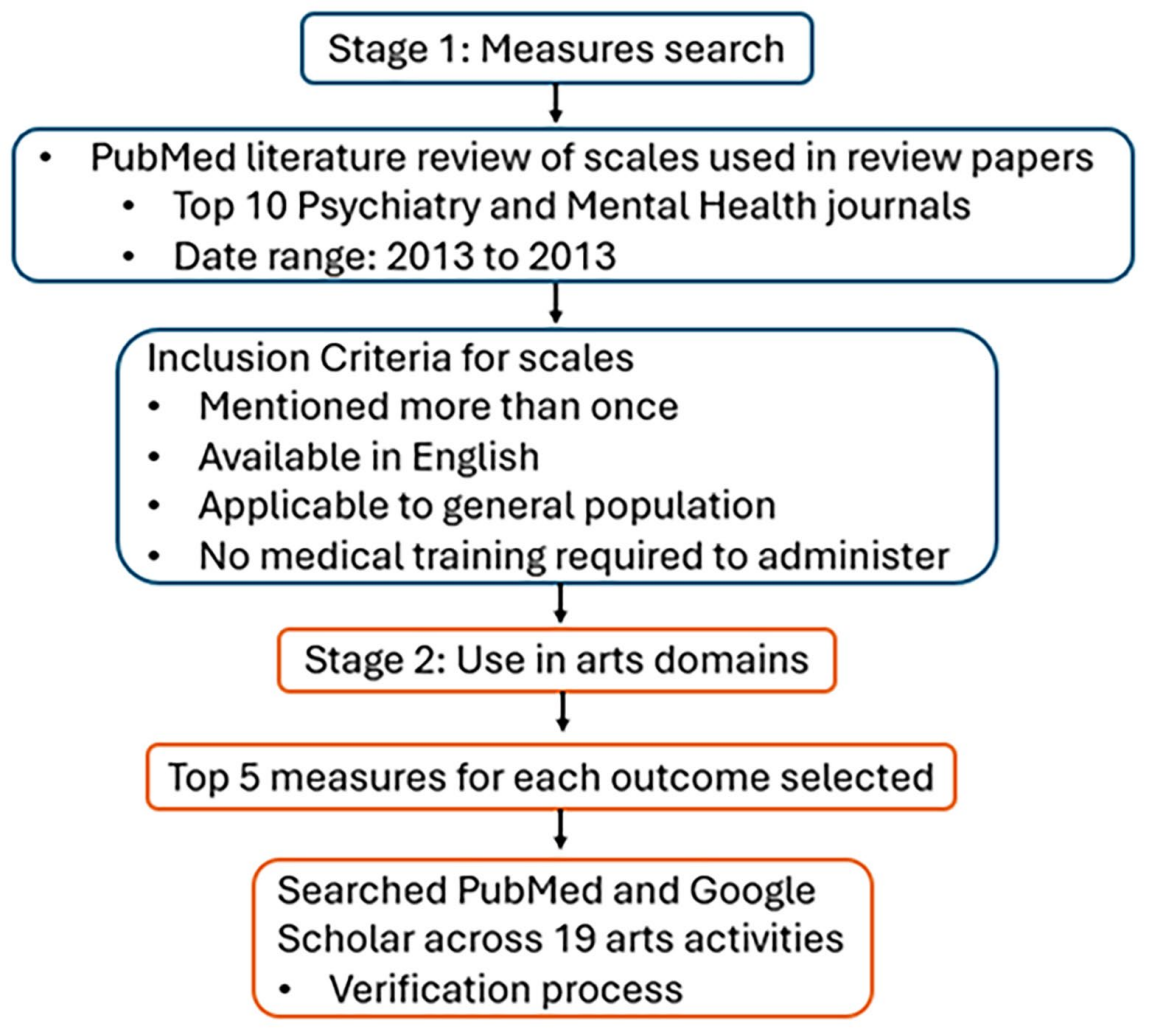
Summary diagram of methods.

At this stage, we also adopted a verification process for the search results of these arts activities to improve the reliability of our results and verify that papers in our searches were accurate and relevant. This process involved searching for irrelevant words commonly associated with art activities to remove irrelevant articles and conducting an extensive manual review of papers in our search results to identify potential false positives (refer to S.1 Verification Process) (Figure 2).

## 3 Results

### 3.1 Depression

#### 3.1.1 Depression review

A total of 46 records were identified from our search, with 25 deemed relevant. From these 25 review papers, 44 scales measuring depression were identified. Of these 44 scales, 24 appeared more than once in the 25 relevant review papers. The frequency of each scale appearing in our literature search can be found in Supplementary material S2, and the frequency of scales appearing more than once is displayed below in Table 1.

Twelve out of 24 depression scales did not meet our inclusion criteria. Additionally, the Brief Symptom Inventory-18 and Kessler-10 were removed, as these scales mainly measure psychological distress (Meachen et al., 2008). However, the BSI-18 appeared in our literature review, with one of its three subscales being depression, while a high score on the K-10 may indicate the presence of a psychological disorder and a possible depression- or anxiety-related mental disorder. The Symptom Checklist-90 was also excluded, as it assesses many symptoms of psychopathology, with depression being one of the nine psychological conditions it evaluates.

The scales were further refined to exclude those specific to a particular setting or demographic. A total of 9 out of the 24 depression scales fit within our inclusion criteria of not requiring a clinician or trained personnel to administer and were applicable for use with the general population. We selected the top 5 of these for further evaluation.

1. Beck Depression Inventory (BDI)
2. Center for Epidemiologic Studies Depression Scale (CES-D)
3. Hospital Anxiety and Depression Scale (HADS)
4. Patient Health Questionnaire-9 (PHQ-9)
5. Depression, Anxiety, Stress Scale-21 (DASS-21)

The five scales have a range of 7–21 items, measure depression, use 4-point scales, take between 5 and 20 min to complete, and four of the five are free to use for non-commercial purposes.

#### 3.1.2 Characteristics of depression scales

Reviewing the frequency of the depression scales listed above allowed us to better narrow down our scope. Nine depression scales met the criteria of being self-administered, appearing more than once in research papers, and being designed to measure depression symptoms.

Characteristics of these depression scales were evaluated based on the following criteria: cost (which indicates whether the scales are copyrighted or in the public domain), time to complete, number of items, levels of measurement, subscales, who the scale is administered by, the scale’s recall period, and its validation in terms of language, culture, and clinical group. These details are provided in Table 1.

Several scales in this list also have subscales that allow one to differentiate between different depressive symptoms. All scales have also been validated in community settings and various clinical groups, and translated into various European and Asian languages. Four scales have a recall period evaluating the past 2 weeks, while one only instructed respondents to recall the past week.

### 3.2 Anxiety

#### 3.2.1 Anxiety review

A total of 38 records were found from our initial literature search, with 15 review papers deemed relevant. A total of 81 scales were extracted from these 15 relevant review papers; however, many scales only measure a singular feature related to anxiety (e.g., rumination, worry, mental pain, and so on) and were excluded from this review. As we aimed to provide arts practitioners with measures of anxiety, we only included scales that were practical measures of anxiety and removed the scales that reported only to measure a singular feature in isolation, as these may also be used as practical indicators of other psychological conditions.

Of the 81 scales, only six met our criteria for having appeared more than once in the list of relevant articles. However, the Penn State Worry Questionnaire (PSWQ) and Brief Symptom Inventory-18 (BSI-18) were not included, as the PSWQ measures worry, and the BSI-18 mainly measures psychological distress (Meachen et al., 2008). The frequency of each scale’s appearance in our literature search can be found in Supplementary material S3.

Although we aimed to shortlist the top five scales for each outcome measure included in this review, we have only identified four anxiety scales that meet our inclusion criteria, which are:

1. Hospital Anxiety and Depression Scale (HADS)
2. Beck Anxiety Inventory (BAI)
3. Generalized Anxiety Disorder Scale (GAD-7)
4. State–Trait Anxiety Inventory (STAI)

#### 3.2.2 Characteristics of anxiety scales

Characteristics of the above scales were evaluated based on the same seven factors mentioned above. Details are compiled below in Table 1.

The anxiety scales we found have a wide range of items, from 7 to 40 in the STAI. However, the STAI is a unique scale in the list, with state and trait anxiety subscales that may not always be used together, depending on a study’s design; thus, only 20 items might be used. All scales have differing recall periods, ranging from current to 2 months. Two scales are free for non-commercial use, and two are copyrighted. All scales in the list have been validated for use in community samples and translated into various European and Asian languages.

### 3.3 Popularity of each depression tool

Among the five shortlisted scales measuring depression symptoms, the BDI was the most popular scale in review papers, followed by the CES-D, the HADS, the PHQ-9, and the DASS-21 (see Table 2). However, this pattern was not observed in our searches across different databases or across the various arts domains included in this review.

**Table 2:** Results of general searches with depression scales.

Scale	BDI	CES-D	HADS	PHQ-9	DASS-21
Appearances*	18	15	12	10	5
PubMed	8,698	2,179	8,439	4,756	2077
Google Scholar	412,000	5,030	252,000	52,600	7,450
HaPI	251	265	100	152	46

*Frequency in the 25 review papers identified in the present search.

In our general searches, the BDI was the most popular in both the PubMed and Google Scholar databases, highlighting its popularity and prominence in medical, public health, and many social science-related studies. On PubMed, the BDI was followed by the HADS, the PHQ-9, the CES-D, and the DASS-21, while on Google Scholar, the popularity of the DASS-21 and the CES-D was flipped. On the HaPI, the CES-D was the most popular scale with a narrow margin over the BDI, followed by the PHQ-9, the HADS, and then the DASS-21.

The BDI was also the most popular scale used among arts-based papers indexed on PubMed, although overall counts for most arts domains were low in general for all scales. The PHQ-9 also yielded more results than the BDI and the HADS in the “arts” domain on PubMed (refer to Supplementary material S4), although this was not observed on Google Scholar.

**Figure 3:**
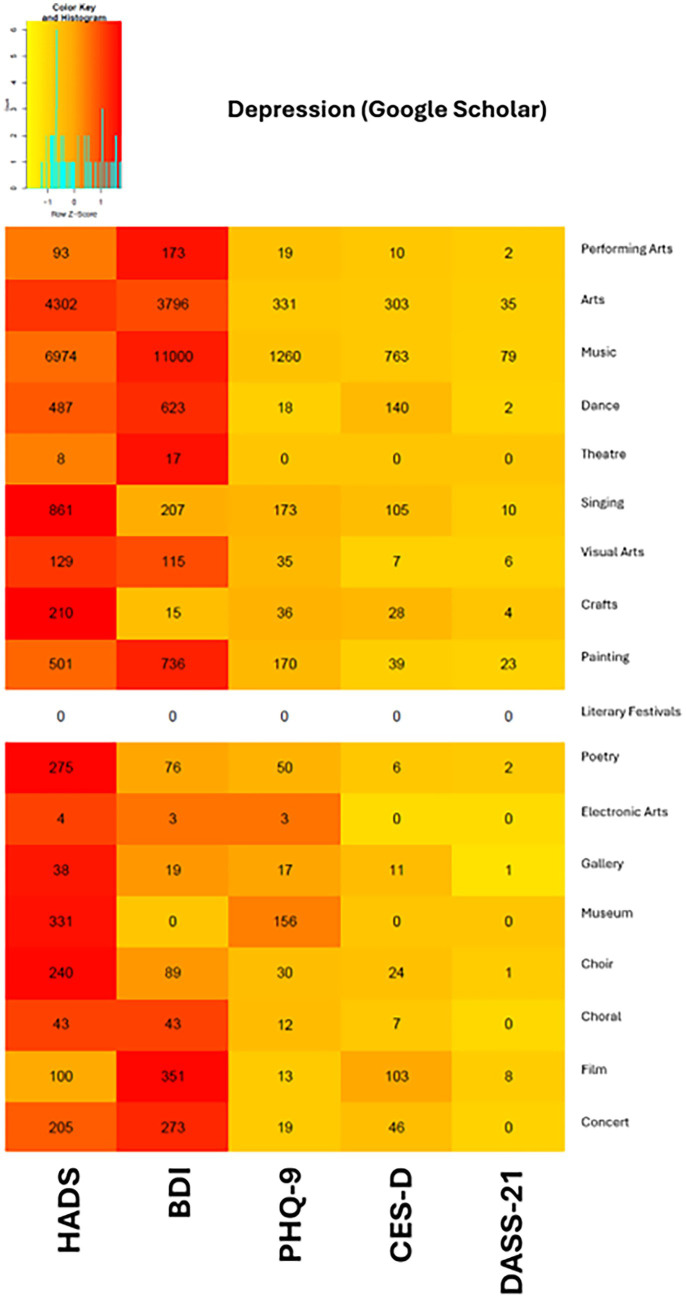
Heat map of depression scales from Google Scholar search results.

The BDI was not consistently the most popular depression tool when searching within arts activities. On Google Scholar, the HADS was found to be more popular than the BDI in nine of the 18 arts domains, while the BDI was more popular than the HADS in eight of the 18 domains (Figure 3).

#### 3.3.1 Use case for depression scale in the arts

The HADS tended to be the popular option when interventions measured both depression and anxiety as their outcomes, perhaps because of its utility in measuring both outcomes with one questionnaire while also being a brief measure with only seven items for each outcome. In papers such as Coulton et al. (2015) study on the effectiveness of a 14-week community group singing program in older adults, the authors utilized the HADS to measure participants’ mental health symptoms. Researchers administered the scale, among other measures, at baseline, 3 months, and 6 months, and found significant reductions in depression among those in the community singing group.

The HADS may not, however, always be the most suitable option across use cases, as it focuses primarily on non-physical symptoms, such as anhedonia, and does not cover the full range of depressive symptoms. For instance, Ugwuanyi et al. (2022) aimed to assess the level of depressive symptoms among parents of children with disabilities. They selected the BDI to capture the breadth and depth of depressive symptoms, which was likely more suitable than the HADS, as the BDI assesses seven diagnostic criteria for depression, whereas the HADS addresses only two. Additionally, the HADS provides a narrower scoring range, with only seven depression items compared to the 21 items included in the BDI.

### 3.4 Popularity of each anxiety tool

For our general search, the HADS was the scale that appeared most frequently on PubMed and Google Scholar, yielding more than double the results on PubMed and more than 50% on Google Scholar compared to the second most popular scale on both databases, which was the STAI for both. On PubMed, the least popular scales were the GAD-7, followed by the BAI, while this was flipped for Google Scholar.

However, we found that the GAD-7 and the BAI yielded more results than the HADS and the STAI on HaPI and appeared more frequently in the review papers from the first part of this study. The BAI was the most popular scale in the review papers, followed by the GAD-7 and the HADS, with the STAI having the least popularity. On the HaPI, the GAD-7 was the most popular scale, followed by the BAI, then the HADS, and the STAI, which had a similar number of results.

Although the GAD-7 was the most popular in the papers we reviewed and on HaPI, it was less popular on PubMed and Google Scholar, with fewer than half the results on PubMed and less than a quarter of the results on Google Scholar compared to the most popular scale. This popularity on HaPI may reflect the GAD-7’s usefulness as a screening tool for generalized anxiety disorder and the three other common anxiety disorders: panic disorder, social anxiety disorder, and post-traumatic stress disorder (Kroenke et al., 2007; Table 3).

**Table 3:** Results of general searches with anxiety scales.

Scale	HADS	BAI	GAD-7	STAI
Appearances*	4	3	3	3
PubMed	8,440	1911	3,455	4,098
Google Scholar	59,700	20,500	17,300	35,500
HaPI	100	121	150	97

*Frequency in the 15 review papers identified in the present search.

**Figure 4:**
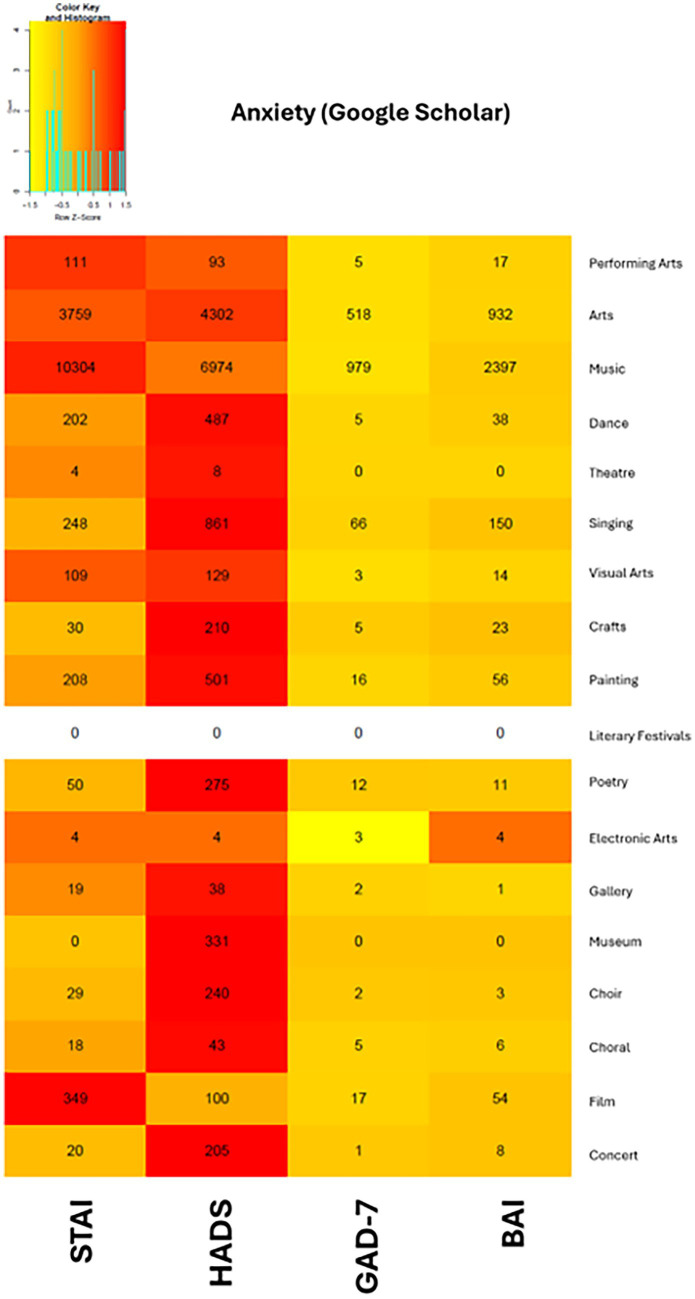
Heat map of anxiety scales from Google Scholar search results.

In our searches within arts domains, the HADS was the overwhelmingly popular option on Google Scholar, with the most results in 13 of the 18 domains, while the STAI was the most popular in four domains. This may be due to the cost associated with using the STAI, which may be prohibitive for some arts practitioners, while the HADS and GAD-7 may be used freely for non-commercial purposes. On PubMed (refer to Supplementary material S5), the STAI was also the most popular option in five domains, while the HADS was the most popular in four domains, although many cells had low counts. However, across PubMed and Google Scholar, the STAI was consistently the most popular scale for the performing arts, music, and storytelling domains (Figure 4).

#### 3.4.1 Use case for anxiety scale in the arts

Galal et al. (2021) pilot study on reducing test anxiety through music interventions provides us with a good illustration of how the STAI can be used in a single-session intervention. During the 2-h-long intervention, an abbreviated 20-item version of the STAI (10 items for state anxiety and 10 items for trait anxiety) was administered three times at pre-, mid-, and post-test. A 25-min passive listening or active playing intervention took place between the mid- and post-test administrations of the STAI.

Notably, especially for those with limited data collection time, the 6-item version of STAI-state (Marteau and Bekker, 1992) is often used instead of the full 20-item state scale. Results from this shortened version produced comparable scores to the full 20-item version, with acceptable reliability and validity, and were found to be suitable for interventions that require a shorter measure of state anxiety.

## 4 Discussion

The first stage of our literature review enabled us to achieve our aims for this paper. We gathered a total of 43 review papers, providing a wide breadth of validated scales that we further investigated in the second stage, examining the arts and health context. Subsequently, the second stage of our literature review generated informative results on the usage patterns of these scales and highlighted their suitability for use in arts-based interventions. We hope that this contribution improves the accessibility of conducting evaluations for artist practitioners and leads to building a stronger evidence base for the effects of the arts on health.

Particularly, music-related art domains, such as “music,” “singing,” “choral,” and “choir,” were highly represented in our literature search. Among all the scales we examined, the HADS appeared to be the most popular choice in arts-based interventions for measuring both depression and anxiety symptoms, as evidenced by its popularity across a large number of art domains included in this paper.

Among scales measuring depression, the HADS and the BDI emerged as the most widely used scales across art domains. The BDI was most popular within the “performing arts,” “music,” “dance,” “theater,” “painting,” “film,” and “concert” domains. Of all the four scales measuring anxiety symptoms, the HADS was more popular in all art domains except “performing arts,” “music,” and “film,” where the STAI was more popular. Nevertheless, we observed a common pattern across all the scales we evaluated in both outcome measures, where no one scale was found to be universally popular in all databases. Even in our searches within the 18 arts domains, no single scale was found to be universally popular across all domains, and this remains true even for scales such as the HADS and DASS-21, which measure symptoms of both outcome measures included in this study.

The popularity of the BDI and the STAI in our general searches and certain arts domains also suggests that cost may not be a strong deciding factor for many users, at least within certain subfields, when selecting a scale. However, many arts practitioners may not have access to or may not have acquired funding and might be limited by the cost of scales in their decision-making process. As such, we have also provided recommendations for arts practitioners who are constrained by a budget that may be freely used for non-commercial purposes.

It is also important to note that the scales highlighted in this paper are not sufficient, without a corresponding clinical assessment, for diagnosis. That is, practitioners cannot use the scales identified in our reviews to *diagnose* individuals—this must be done in a clinical setting by a clinician, as diagnoses may have significant impacts on an individual’s own perception of the state of their health and possibly other social, financial, and professional implications.

### 4.1 Context of non-clinical arts-based interventions

Research in the context of arts-based interventions contains several differences that make them different and novel compared to traditional medical and clinical interventions (Ike and Howell, 2022). Moreover, the most obvious difference is the settings and venues in which arts interventions often occur. Many interventions, including arts-on-prescription interventions that flow down from healthcare providers, take place in community venues such as concert halls or theaters, community centers, museums and galleries, dance and music studios, and even open public spaces such as festival grounds. In contrast, hospitals, laboratories, and primary care settings where medical research traditionally takes place are well-staffed, better controlled, and have spaces that can be dedicated to data collection.

Next, although arts-based programs may have multi-session designs similar to many clinical trials, many arts programs may struggle to design programs with multiple data collection windows. Many arts programs have short-term contact with their participants, typically lasting just an hour. They thus may only expect to have an impact on short-term aspects of mental health, such as anxiety symptoms or stress (which was not within the scope of this review), rather than depressive symptoms.

It is also important to note that, as opposed to emotional states—which may change momentarily (and as a function of participating in a single arts event)—certain outcome measures, such as the measures of mental health discussed here, would not normally be expected to change as a function of an *individual* arts/cultural session. Typically, longer interventions (there is a great range in the literature) are required to have a significant and lasting impact on mental health. Therefore, if the artist practitioner intends to impact depression or anxiety symptoms, they should plan for repeated sessions rather than individual sessions. As an example, while it may be appropriate to use the State portion of the STAI in a pre- and post-test of a single session, it would not be appropriate to use the Trait portion, which is based on long-term features of one’s personality/disposition. Therefore, it is important to consider the context and reasonably expected results of interventions; individual sessions are not as likely as months-long interventions to have a significant impact on mental health (Quinn et al., 2025).

### 4.2 Recommendations for practitioners

Again, these scales have been validated in public health contexts and are appropriate for the wide range of community members for whom artists perform. They are not necessarily tailored for specific clinical populations (e.g., those diagnosed with dementia), children, or seniors. However, the questionnaires mentioned in this paper may also have been validated in certain clinical populations. These public health contexts refer to use with a general adult population in community settings.

Though the most popular scales in existing published arts-related studies were the HADS and the STAI for depression and anxiety, respectively, practitioners need to consider various other factors besides the popularity of a specific scale.

For instance, despite the popularity of the HADS and its ability to measure both depression and anxiety, it only focuses on a subset of the symptoms of each disorder. On the other hand, the PHQ-9 covers a broader view of depression, with each item covering one symptom that corresponds with one of the nine diagnostic criteria, while the BDI provides a more comprehensive view with 21 items and is able to more sensitively determine the severity of symptoms. Ideally, practitioners should identify the specific symptoms their programs target and choose a scale that aligns with these goals.

Another point of consideration is the practitioner’s intervention design, the recall period of the scale, and the number of times it will be administered. As mentioned above, interventions consisting of just a single session will likely be limited in the level of expected change they can influence (Kim et al., 2023). As such, the options for validated scales with suitable recall periods for a pre- and post-test intervention consisting of a single session are also limited.

**Figure 5:**
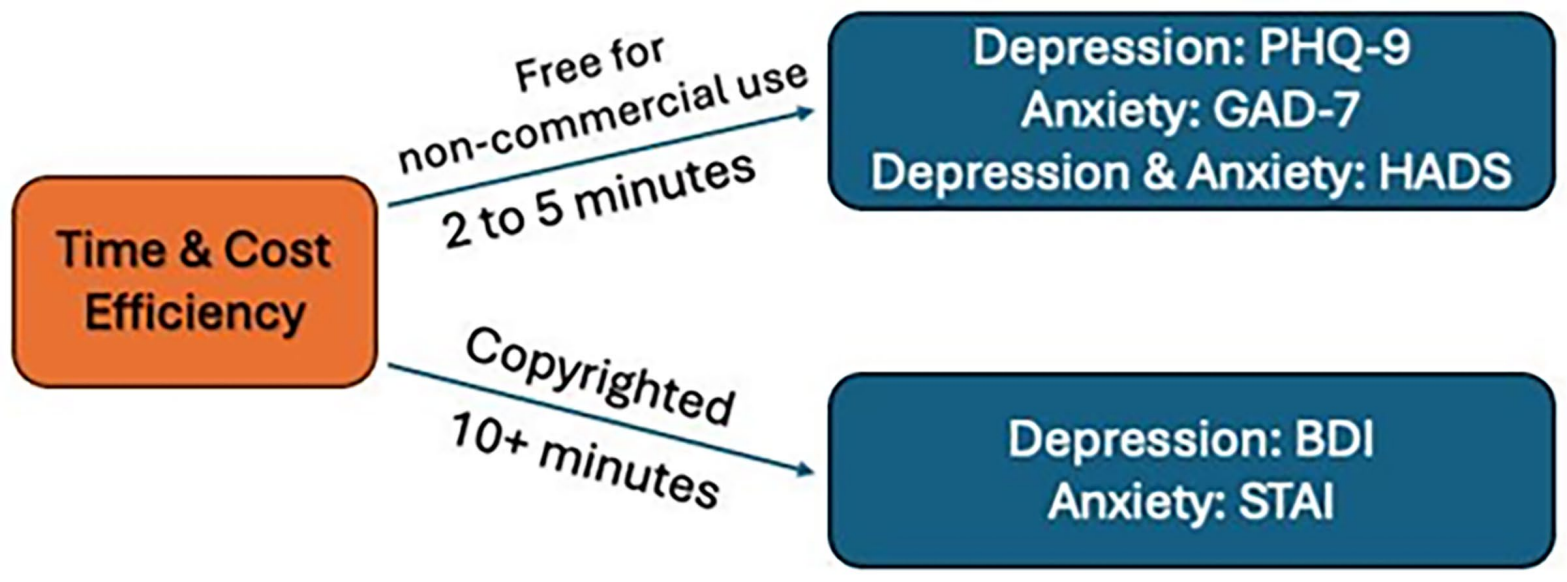
Simple considerations when selecting evaluation tools.

**Figure 6:**
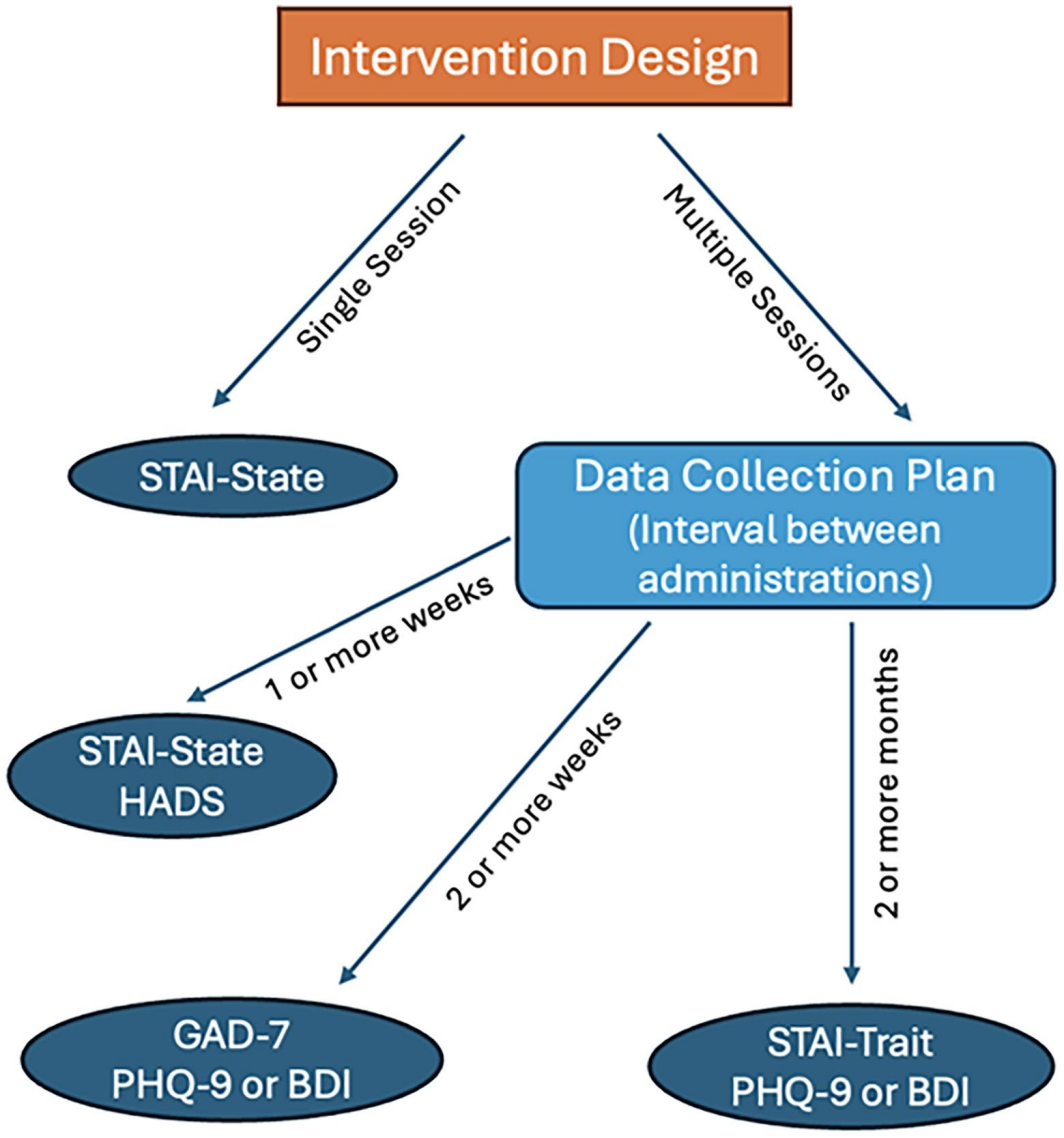
Simple selection of evaluation tools according to evaluation design.

Figure 5 illustrates some basic considerations that artist practitioners should keep in mind when selecting their evaluation tools, while Figure 6 illustrates how program schedules or intervention design can guide their choice of evaluation tools.

## 5 Conclusion

We provided a two-step literature review that explored the publication landscape of the medical, public health, and arts literature. We first identified the most reviewed validated scales in various domains of published scientific literature in medical and public health databases. Using this methodology, we compiled a set of scales used to measure symptoms within two outcomes: depression and anxiety.

Thereafter, we selected the five most popular scales used to measure each outcome, explored their frequency of use across 18 arts domains, and examined their suitability and ease of use for arts and health practitioners who wish to evaluate the effect of their activities on their target audience’s mental health. As we seek to increase the accessibility of evaluation in the arts and health field, we also provide recommendations to simplify the decision-making process for practitioners, with the goal of strengthening the evidence base for the field.

Our study differs from previously published toolkits and frameworks in the arts and health field by specifically highlighting scales that measure symptoms of depression and anxiety, providing a clear rationale and methodology for these recommendations, and offering guidance tailored to common contexts and program designs in which arts interventions are delivered.

### 5.1 Limitations

A limitation of this study pertains to the use of databases for our popularity search. We identified several irrelevant articles in both databases and addressed this issue by examining the first 50 results in each search to determine the false positive rate, which we then used to adjust the number of results for each search individually. Although this method may not provide us with exhaustively accurate numbers of relevant papers for every arts domain and scale, we believe that it provides a good depiction of the state of the field, as well as the outcome measures and scales used within different arts domains.

Though we aimed to provide a top five list of scales for each outcome measure we examined in this study, our inclusion criteria—specifically the criterion that a scale appear in at least two review papers—were not met, despite the 18 review papers we included for anxiety, as only four scales appeared in more than two review papers. Additionally, existing literature has highlighted a prior lack of brief and self-reported validated measures of anxiety (Spitzer et al., 2006), which inspired the development of the GAD-7. However, although the GAD-7 may have addressed the gap in clinical practice, as observed in its popularity on HaPI, the same gap may still exist in research, as seen in our review of the existing literature, where the GAD-7 falls short of all three other scales on Google Scholar and behind the HADS and the STAI on PubMed. Finally, the arts are an incredibly rich and diverse medium, with greatly varying goals and program structures for each art form. As such, we were only able to provide recommendations for more commonly used program structures, taking into account the more common considerations and restraints that artist practitioners typically face. However, we hope that the thought processes and principles laid out may also ease the decision-making process for artist practitioners wishing to evaluate their programs.

### 5.2 Future research

Others in the field (Smith et al., 2021) have made a “call to action” for a core outcome set to be agreed upon that is relevant and specific to the field of arts and health, similar to the core outcomes in Social Prescribing (Sonke et al., 2023). The purpose of creating a core outcome set is complementary to our goal in this paper, which is to drive the field forward by improving the evidence base for the effects of the arts on health. While a core outcome set aims to identify a range of outcomes of interest for the field, the goal of this study is to identify the actual scales that may be employed for investigating such outcomes.

By exploring the most popular scales in commonly examined outcomes of interest to the field of Arts & Health, such as depression and anxiety, we enable and empower arts and health practitioners to be better equipped to conduct more rigorous evaluation and research. Therefore, future work should move along three complementary pathways.

First, we concur with others that future studies should aim to formulate and agree on a core outcomes set, which includes health outcomes that may reasonably be impacted through arts engagement. Second, to increase the accessibility of evaluation and research, future research may also examine the most popular scales in a range of other outcomes, such as quality of life and social connection, to build up a toolbox of suggestions and recommended scales that suit the unique characteristics of the field. Third, future research within the arts and health field may aim to expand on this study by identifying and recommending outcome measures for use in arts interventions with specific populations, such as pediatric, adolescent, older adult, or large clinical populations. These complementary pathways would provide arts practitioners and researchers with a common goal of improving health and a set of measurement tools that can be used to investigate these outcomes.
