# Supplementary material for: Identifying and recommending validated measures to assess depression and anxiety outcomes in the field of arts and health
Source: Front Psychol. 2025 Sep 3;16:1628292. doi: 10.3389/fpsyg.2025.1628292 (PMC12442041; doi:10.3389/fpsyg.2025.1628292)

Supplementary Materials

S.1 Verification Process

We identified exclusion terms by combing through the first 50 results for the two most popular scales for each outcome measure and searching for wherever the term of interest occurred. This was done for four domains (arts, dance, theatre, and film) which we found to have leakage from other adjacent terms or were portions of other common words (such as “parts”, “accordance”, “operating theatre”, and “film-coated”). When an exclusion term was identified, the term was added iteratively to the search algorithm to exclude it from the search (e.g., NOT “parts” in Pubmed and –“parts” in Google Scholar) and this was repeated until up to 50 papers were examined.

For verification of the relevance of papers identified in the searches, we reviewed the first 50 papers in every search (within each arts domain), or all papers for searches that yielded fewer than 50 results. This method was adopted, as it was not feasible to review every paper in all of our searches, as some search results numbered in the thousands, nor was it possible to draw a random sample from Google Scholar results. From this process, we generated a ‘false positive rate’ for each search, and used this rate to adjust the number of results from each respective search. This was done by using the false positive rate and subtracting it from the number of results of the corresponding search.

S.2 List of All Depression Scales that Appeared in our Review

|  | **Scale** | **Frequency** |
| --- | --- | --- |
| 1 | Beck Depression Inventory | 18 times |
| 2 | Center for Epidemiologic Studies Depression Scale | 15 times |
| 3 | Hospital Anxiety and Depression Scale | 12 times |
| 4 | Hamilton Depression Rating Scale | 12 times |
| 5 | Patient Health Questionnaire-9 | 11 times |
| 6 | Brief Symptom Inventory - 18 | 7 times |
| 7 | Montgomery-Asberg Depression Rating Scale | 7 times |
| 8 | Geriatric Depression Scale - 15 | 6 times |
| 9 | Depression, Anxiety, Stress Scale-21 | 5 times |
| 10 | Zung Depression Self-Rating Scale | 4 times |
| 11 | Symptom Checklist-90 | 4 times |
| 12 | Structured Clinical Interview for DSM | 4 times |
| 13 | Edinburgh Postnatal Depression Scale | 3 times |
| 14 | Quick Inventory of Depressive Symptomatology Self-Report | 3 times |
| 15 | Composite International Diagnostic Interview | 3 times |
| 16 | Child Depression Rating Scale Revised | 3 times |
| 17 | Kiddie Disorders for Affective Disorders Scale | 3 times |
| 18 | Beck Depression Inventory - Short Form | 2 times |
| 19 | Edinburgh Depression Scale | 2 times |
| 20 | Kessler-10 Questionnaire | 2 times |
| 21 | Mini International Neuropsychiatric Interview | 2 times |
| 22 | KSADS-PL (Present and Lifetime) | 2 times |
| 23 | Profile of Mood States - 40 | 2 times |
| 24 | Brief Psychiatric Rating Scale | 2 times |
| 25 | Brief Symptom Inventory | 1 time |
| 26 | Brief Edinburgh Depression Scale | 1 time |
| 27 | General Health Questionnaire - 12 | 1 time |
| 28 | General Health Questionnaire - 28 | 1 time |
| 29 | Neuropsychiatric Inventory (NPI) | 1 time |
| 30 | Cornell Scale for Depression in Dementia | 1 time |
| 31 | Comprehensive Psychopathological Rating Scale | 1 time |
| 32 | Cambridge Mental Disorders for the Elderly Examination | 1 time |
| 33 | Primary Care Evaluation of Mental Disorders (PRIME-MD) | 1 time |
| 34 | Self-rating Depression Scale | 1 time |
| 35 | Inventory to Diagnose Depression | 1 time |
| 36 | Mini Mood and Anxiety Symptom Questionnaire | 1 time |
| 37 | Psychiatric Epidemiology Research Interview | 1 time |
| 38 | Affects Balance Scale | 1 time |
| 39 | Brief Psychiatric Rating Scale - Expanded | 1 time |
| 40 | Depressive Experiences Questionnaire | 1 time |
| 41 | Major Depressive Episode Screener | 1 time |
| 42 | Reynolds Adolescent Depression Scale - Second Edition | 1 time |
| 43 | Depression Severity Rating | 1 time |
| 44 | KSADS-E (Epidemiological) | 1 time |

S.3 List of All Anxiety Scales that Appeared in our Review

|  | **Scale** | **Frequency** |
| --- | --- | --- |
| 1 | Hospital Anxiety and Depression Scale (HADS) | 4 times |
| 2 | Penn State Worry Questionnaire (PSWQ) | 4 times |
| 3 | State-Trait Anxiety Inventory (STAI) | 3 times |
| 4 | Beck Anxiety Inventory (BAI) | 3 times |
| 5 | Generalized Anxiety Disorder Scale (GAD-7) | 3 times |
| 6 | Brief Symptom Inventory-18 | 2 times |
| 7 | Depression, Anxiety, Stress Scale (DASS-21) | 1 time |
| 8 | Hamilton Anxiety Rating Scale (HAM-A) | 1 time |
| 9 | Death Anxiety Scale | 1 time |
| 10 | Death Anxiety Inventory | 1 time |
| 11 | Death Anxiety Questionnaire | 1 time |
| 12 | Liebowitz Social Anxiety Scale | 1 time |
| 13 | Geriatric Anxiety Inventory (GAI-SF) | 1 time |
| 14 | Post Traumatic Stress Disorder Checklist 17 | 1 time |
| 15 | Hopkins Symptom Checklist-25 | 1 time |
| 16 | Post Traumatic Stress Disorder Checklist Civilian Version | 1 time |
| 17 | Brief Symptom Inventory-3 | 1 time |
| 18 | Harvard Trauma Questionnaire | 1 time |
| 19 | DSM-IV 16 item derived criteria for PTSD | 1 time |
| 20 | Mental Pain Questionnaire | 1 time |
| 21 | Psychological Pain Assessment Scale | 1 time |
| 22 | Psychache Scale | 1 time |
| 23 | Orbach and Mikulincer Mental Pain Scale | 1 time |
| 24 | Tolerance for Mental Pain Scale | 1 time |
| 25 | Mee-Bunney Psychological Pain Assessment Scale | 1 time |
| 26 | Pictorial Representation of Illness and Self Measure | 1 time |
| 27 | Panic Disorder Severity Scale (PDSS) | 1 time |
| 28 | Cognitive Avoidance Questionnaire | 1 time |
| 29 | Attentional Control Scale | 1 time |
| 30 | Contrast Avoidance Questionnaires | 1 time |
| 31 | Anxiety Disorders Interview Schedule | 1 time |
| 32 | Collett-Lester Fear of Death Scale | 1 time |
| 33 | Multidimensional Fear of Death Scale | 1 time |
| 34 | Ruminative Response Scale (RRS) | 1 time |
| 35 | Children's Ruminative Response Scale (CRRS) | 1 time |
| 36 | Adapted Ruminative Response Scale (aRRS) | 1 time |
| 37 | Rumination-Reflection Scale (R-RS) | 1 time |
| 38 | Self-Reflection and Self-Rumination Scale (SRuS, SReS) | 1 time |
| 39 | Rumination Scale (RS) | 1 time |
| 40 | Cognitive Emotion Regulation Questionnaire (CERQ) | 1 time |
| 41 | Scott-McIntosh Rumination Inventory (SMRI) | 1 time |
| 42 | Ruminative Thought Style Questionnaire (RTSQ) | 1 time |
| 43 | Thoughts Questionnaire (TQ) | 1 time |
| 44 | Rumination Questionnaire (RQ) | 1 time |
| 45 | Anxious Rumination Questionnaire (ARQ) | 1 time |
| 46 | Post-Event Processing Questionnaire-Revised (PEPQ-R) | 1 time |
| 47 | Penn State Worry Questionnaire-Children | 1 time |
| 48 | Cambridge Worry Scale | 1 time |
| 49 | Brief Measure of Worry Severity | 1 time |
| 50 | Thought Control Questionnaire | 1 time |
| 51 | Worry Domains Questionnaire (TCQ) | 1 time |
| 52 | Adapted Worry Domains Questionnaire (aWDQ) | 1 time |
| 53 | Anxious Thoughts Inventory | 1 time |
| 54 | Worry Survey (WS) | 1 time |
| 55 | Revised Children's Manifest Anxiety Scale | 1 time |
| 56 | "Things I Worry About" Scale (TIWA) | 1 time |
| 57 | Student Worry Scale (SWS) | 1 time |
| 58 | Insomnia Daytime Worry Scale (IDWS) | 1 time |
| 59 | Illness Attitudes Scale (IAS) | 1 time |
| 60 | Event Related Rumination Inventory (ERRI) | 1 time |
| 61 | Response to Intrusions Questionnaire (RIQ) | 1 time |
| 62 | Volitional Components Inventory (VCI) | 1 time |
| 63 | Self-Consciousness Scale (SCS) | 1 time |
| 64 | Response to Positive Affect (RPA) | 1 time |
| 65 | Response to Stress Questionnaire (RSQ) | 1 time |
| 66 | Stress Reactive Rumination Scale (SRRS) | 1 time |
| 67 | Stress Arousal Scale-4 (SAS4) | 1 time |
| 68 | Self-Reflection and Insight Scale (SRIS) | 1 time |
| 69 | Self-Reflection and Insight Scale-Youth (SRIS-Y) | 1 time |
| 70 | Emotion Control Questionnaire (ERQ) | 1 time |
| 71 | Sad and Angry Rumination Inventory (SARI) | 1 time |
| 72 | Angry Rumination Scale (ARS) | 1 time |
| 73 | Rumination about the transgression scale (RATTS) | 1 time |
| 74 | Rumination about an Interpersonal Offence (RIO) | 1 time |
| 75 | Inhibition-Rumination Scale (I-RS) | 1 time |
| 76 | Post-work rumination scale (PWRS) | 1 time |
| 77 | Perseverative Thinking Questionnaire (PTQ) | 1 time |
| 78 | Repetitive Thinking Questionnaire (RTQ) | 1 time |
| 79 | Profile of Mood States-40 (POMS) | 1 time |
| 80 | Five-Facet Mindfulness Questionnaire (FFMQ) | 1 time |
| 81 | Behavioral Activation System (BAS) | 1 time |

S.4 Heat Map of Depression Scales from PubMed Search Results


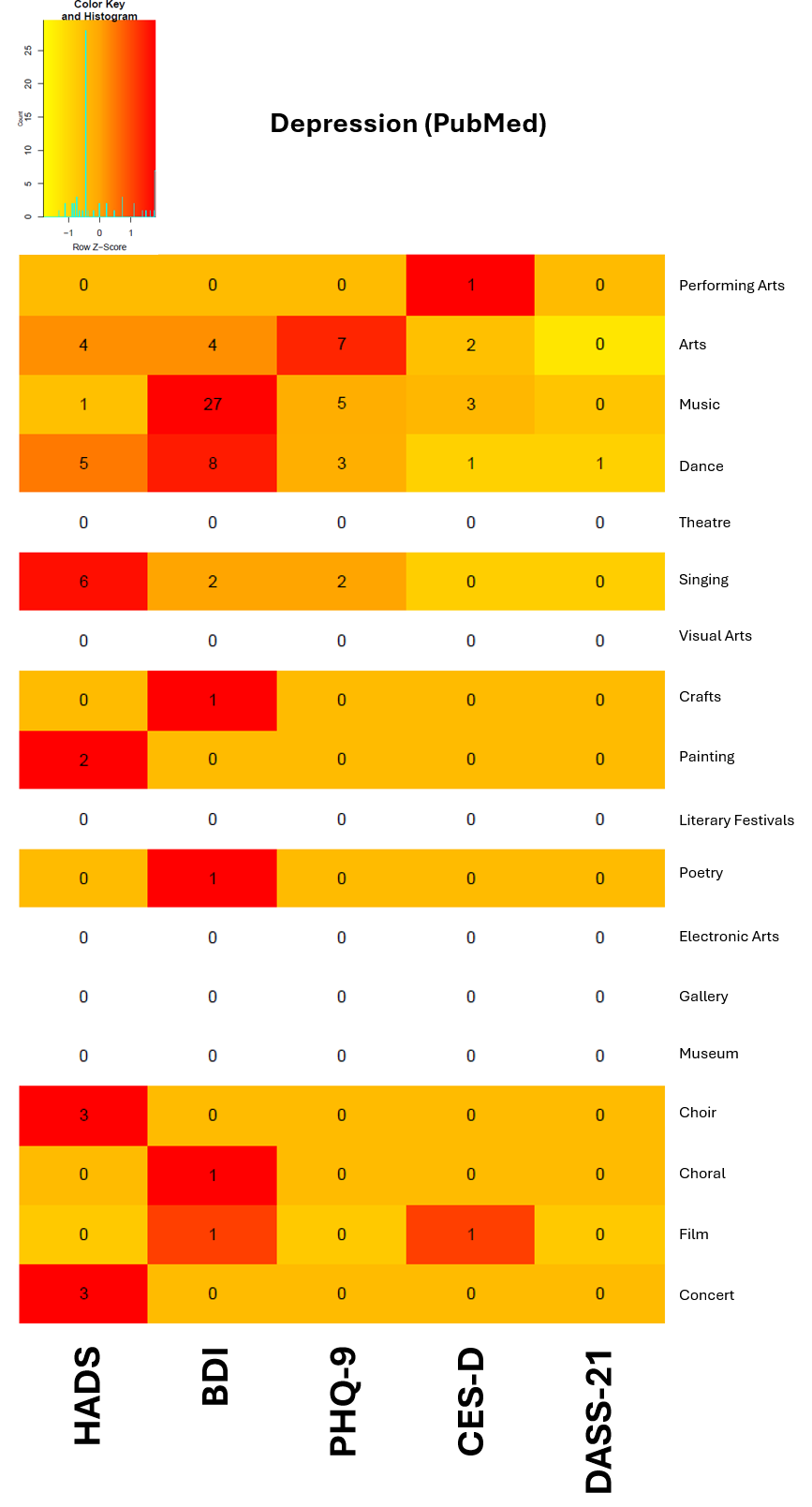


S.5 Heat Map of Anxiety Scales from PubMed Search Results


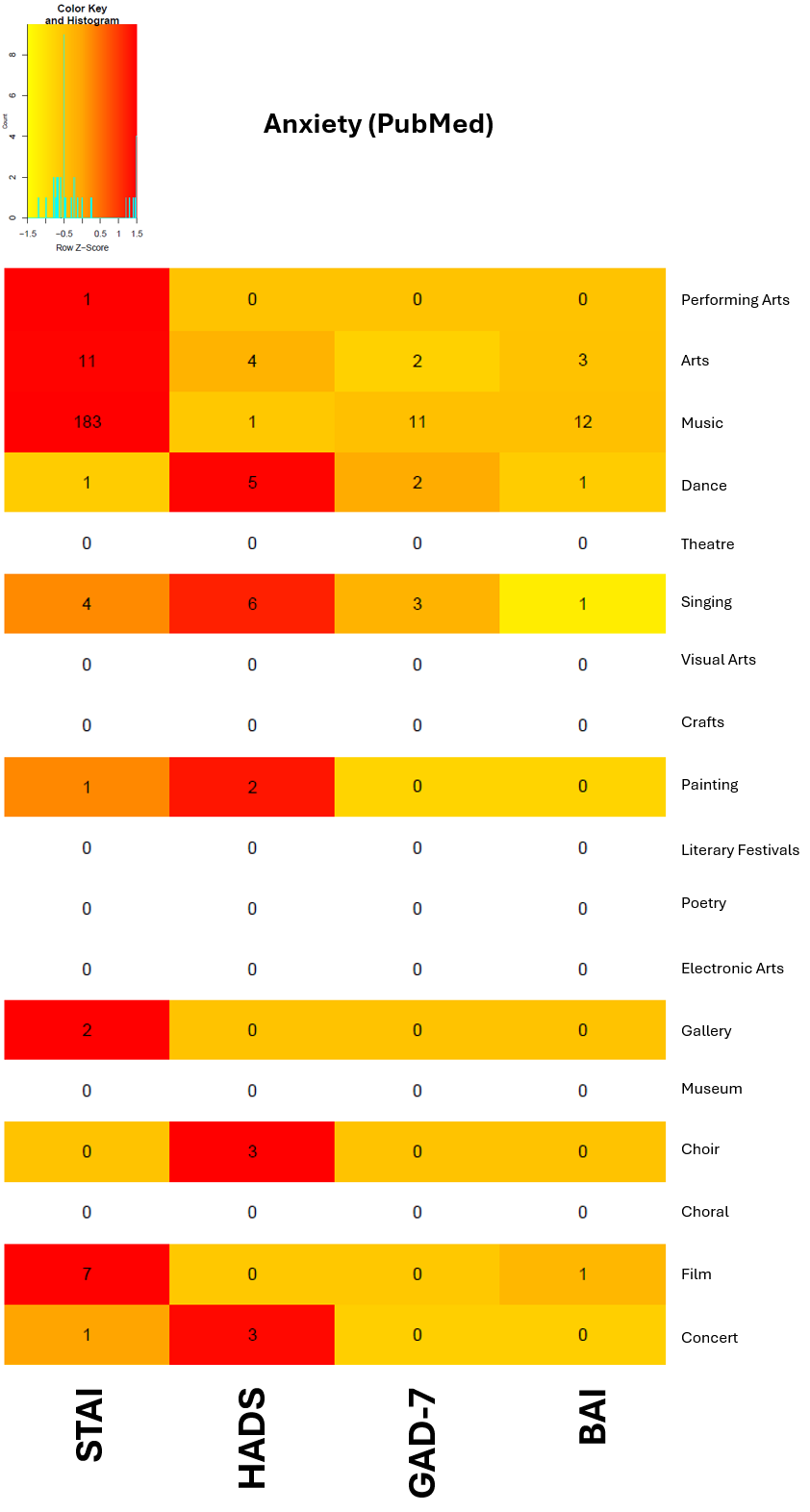

Supplement: Supplementary file 1 [file Supplementary_file_1.docx]
